# A microalga, *Euglena tuba* induces apoptosis and suppresses metastasis in human lung and breast carcinoma cells through ROS-mediated regulation of MAPKs

**DOI:** 10.1186/s12935-016-0330-5

**Published:** 2016-06-29

**Authors:** Sourav Panja, Nikhil Baban Ghate, Nripendranath Mandal

**Affiliations:** Division of Molecular Medicine, Bose Institute, P-1/12, C. I. T. Scheme, VII M, Kolkata, 700054 India

**Keywords:** Microalga, ROS, Antioxidants, MAPK, Anticancer, SOD, DNA binding, Protein binding

## Abstract

**Background:**

*Euglena tuba*, a microalga, is known for its excellent antioxidant and iron-chelation activities; however its anticancer efficacies have not been reported yet. This study investigates the antitumor and antimetastatic activities of 70 % methanolic extract of *Euglena tuba* (ETME) against human lung (A549) and breast cancer (MCF-7) cells in vitro. Moreover, we had examined ETME’s role in inducing intracellular ROS with the regulation of antioxidants and MAPK pathway.

**Methods:**

Anticancer activity of ETME was thoroughly studied using flow cytometry, confocal microscopy and western blotting; along with various biochemical assays for analysing ROS-induced regulation of antioxidant enzymes. Inhibition of invasion and migration of malignant cells by ETME were investigated by wound healing and zymographic studies. DNA–Protein interaction with ETME was also studied.

**Results:**

ETME inhibited the growth of both A549 (IC_50_ 92.14 µg/ml) and MCF-7 cells (IC_50_ 50.27 µg/ml) by inducing apoptosis, while remained non-toxic against nomral WI-38 cells (IC_50_ 911.43 µg/ml). ETME treatment resulted in increasing Bax/Bcl-2 ratio, BID truncation and activation of caspase cascade. This ultimately leads to PARP degradation and apoptosis through the intrinsic and extrinsic pathway in both A549 and MCF-7 cells. Wound healing and gelatin zymography studies revealed that ETME significantly inhibited the invasion and migration of both A549 and MCF-7 cells dose-dependently through the downregulation of MMP-9. Further investigations showed that ETME selectively induces intracellular ROS, regulated the levels of intracellular antioxidants and suppresses the activation of ERK1/2, JNK, P38 mitogen-activated protein kinase pathways in both type of malignant cells. Further DNA and protein binding studies revealed that ETME strongly interact with DNA as well as protein attributing the possibilities of presence of components which are targeting the macromolecules in cancer cells. Moreover, when the identified compounds from ETME were examined for their cytotoxicities individually, it was found that they lost their specificities towards cancer cells and also attacked normal cells.

**Conclusions:**

Our study suggests that ETME retards the growth of both lung and breast cancer cells, in vitro, through multivariate mechanisms, proving its candidature for the development of better and safer drugs against these cancers.

## Background

In the last era, many diseases have overcome through the significant improvement of biomedical science. However, cancer remains elusive, especially from a therapeutic viewpoint. Currently, cancer is treated using radiotherapy, chemotherapy and surgery that are related with severe side effects [1]. Even a large number of tumours are insufficiently responsive to cancer therapeutic drugs and radiotherapy due to multi-drug resistance. Epidemiologic studies suggests that in dietary supplement, increasing consumption of fruits, vegetables, whole grains and related lifestyles is a practical approach for significantly reducing the incidence of cancer. Numerous individual phytochemicals (>5000) are isolated from the plant-based foods [2]. Before we can fully understand the health benefits in humans, their identification, characterization and mechanism of action need to be determined. In mammalian cells, biological targets of phytochemicals were found to be involved in antiproliferative, anti-inflammatory, antiangiogenic, antimetastatic and pro-apoptotic effects, or the ability to reduce oxidative stress [3, 4]. In vitro studies on different cancer cell lines proved the role of polyphenols as growth inhibitors, either by induction of G1-cell cycle arrest [5], G2/M arrest [6], or cell death [7]. Similarly, different carotenoids demonstrated G-1 arrest [8] and apoptosis [9] in various cancer cells. So, identification and development of natural resources used for cancer treatment have attracted a lot of attention globally.

Reactive oxygen species (ROS), a regular by-product of the normal breakdown of oxygen, play a significant role in normal biochemical functions and abnormal pathological processes [10]. There is a subtle balance between intracellular ROS and antioxidant capacity in normal cells, which determines their destiny [11]. Yet cancer cells are characterised by elevated intracellular ROS stress, resulting from carcinogenesis stimulation, abnormal metabolic activity and mitochondrial malfunction. The limited capacity of tumor cells to deal with the elevated ROS levels makes them vulnerable to oxidative stress [12]. This embarks a novel strategy for cancer therapy to promote apoptosis in cancer cells by eliminating the ability of antioxidant defence systems through inducing ROS production [13].

*Euglena tuba* (Carter) (Family, Euglenaceae) is one of the most widespread microalga in the Rarh region of West Bengal, India. Cells of *E.tuba* are normally green, having periplast without spiral rows of granules and more than one chloroplast. Paramylon bodies are present but margins not convolute, with cylindrical and highly metabolic cells that constantly change shapes in movements as they have stiff blue pellicle outside the cell membrane that are flexible in nature [14]. Euglena mainly grows in aquatic bodies with algal bloom all over the year mostly in winter. Various *Euglena* sp. have a broad range of medicinal properties, such as antimicrobial, anti-mutagenic, anti-HIV, immunopotentiating and antitumor activity [15–20] with numerous isolated bioactive compounds such as, vitamin C, vitamin E and β-carotene that can be harnessed for commercial use [21]. In our previous study, we reported that *E. tuba* possess potent in vitro antioxidant and in vivo iron chelation activity [22, 23] and numerous phytochemicals such as phenolics, flavonoids, alkaloids, tannins, terpenoids, triterpenoids, saponin, glycoside and carbohydrates are present in adequate amount in the extract which was confirmed by phytochemical analysis [22]. In this study, we first demonstrated the antiproliferative effect of 70 % methanolic extract of *E. tuba* (ETME) on lung and breast cancer cells and normal fibroblast cells in vitro. Moreover, we have studied that ROS accumulation caused by ETME leads to the activation of apoptosis and inhibition of metastasis through regulation of MAPK pathways.

## Results

### ETME has antiproliferative activity against cancer cells, not in normal cells

**Fig. 1:**
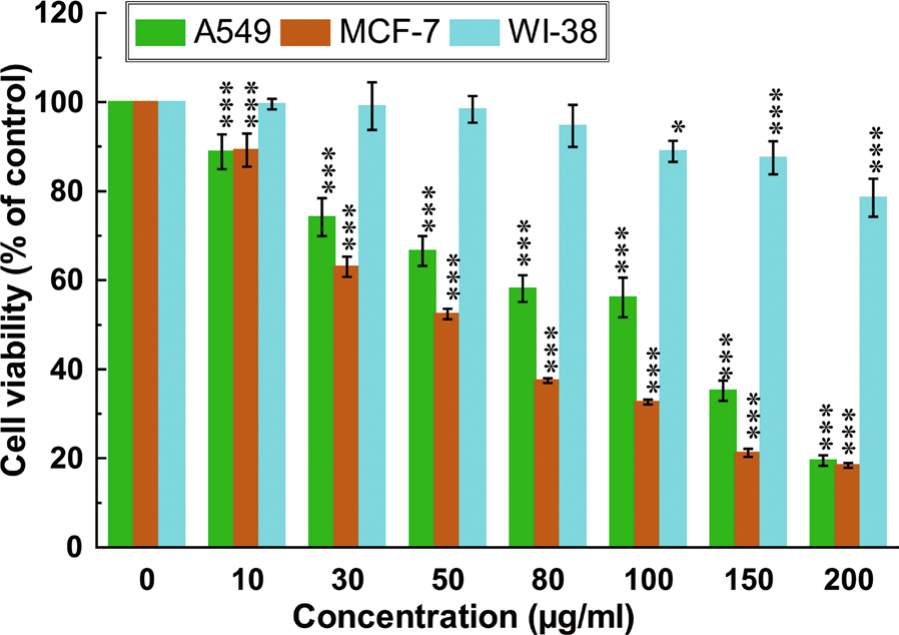
Effect of ETME on cell viability and growth of *A549*, *MCF-7* and *WI-38* cells. WST-1 assay of all cells treated with different concentrations of ETME and incubate for 48 h. Results were expressed as cell viability (% of control). All data is expressed as mean ± SD (n = 6). *p < 0.05 and ***p < 0.001 vs. 0 µg/ml

Cytotoxic effect of ETME was investigated on lung (A549) and breast (MCF-7) carcinoma and also on one non-malignant cell line (WI-38) using WST-1 assay. As shown in Fig. 1, ETME was found cytotoxic against both A549 and MCF-7 cells and inhibited their growth dose-dependently with an IC_50_ value of 92.14 and 50.27 µg/ml respectively. However, the treatment of ETME did not significantly inhibit the cell propagation of WI-38 cells which is corroborated by high IC_50_ value (911.43 µg/ml).

### Cell cycle analysis by flow cytometry

**Fig. 2:**
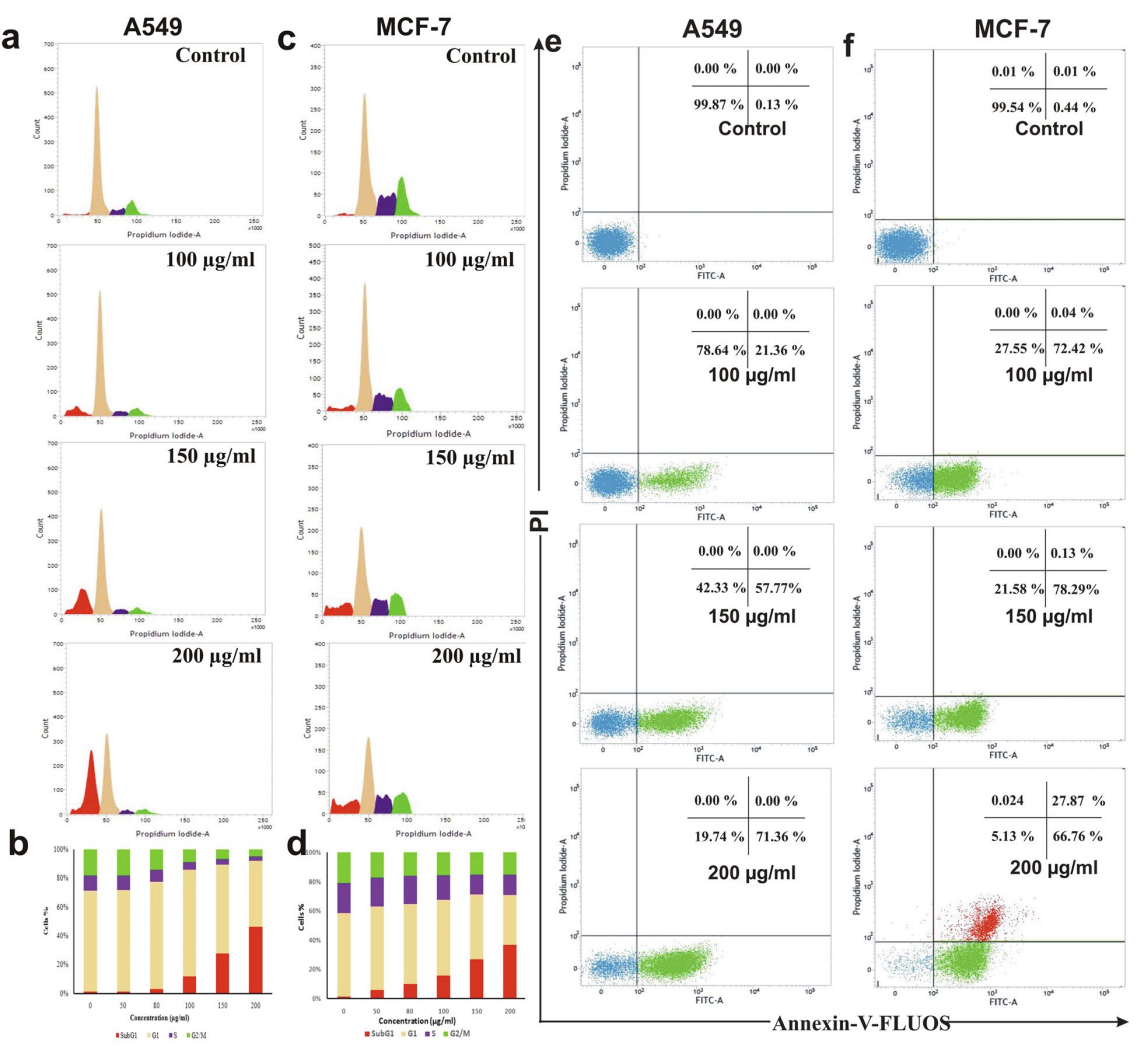
Flow cytometric analysis of *A549* and *MCF-7* cells treated with ETME (0–200 µg/ml). **a** Cell cycle phase distribution of *A549* cells treated with indicated doses for 48 h, **b** Graphical representation of  % cell population in all phases (*Sub-G1*, *G1*, *S*, and *G2/M*) for section A, **c** The phase of cell cycle distribution of MCF-7 cells after ETME treatment with indicated doses for 48 h, **d** Graphical representation of  % cell population in all phases (*Sub-G1*, *G1*, *S*, and *G2/M*) for section B, **e** Apoptosis detection of *A549* cells treated with indicated doses for 48 h, **f** Apoptosis detection of *MCF-7* cells treated with indicated doses for 48 h

The effect of ETME on cell cycle distribution of A549 and MCF-7 cells was studied. Figure 2a–d show that ETME has capablity to induce cell death in A549 cells at 200 µg/ml and MCF-7 cells at 150 µg/ml concentration. After treatment with ETME, the populations in every phase (sub-G1, G1, S, G2/M) were quantified and plotted with increasing doses. It is found that, nearly 33.88 and 22.68 % cells were accumulated in Sub-G1 phase (apoptotic phase) in A549 (200 µg/ml) and MCF-7 (150 µg/ml) cells respectively. This increasing Sub-G1 phase also coincides with the decrease in the cell population of other phases.

### ETME induces apoptosis by Annexin V/PI study and DAPI staining

**Fig. 3:**
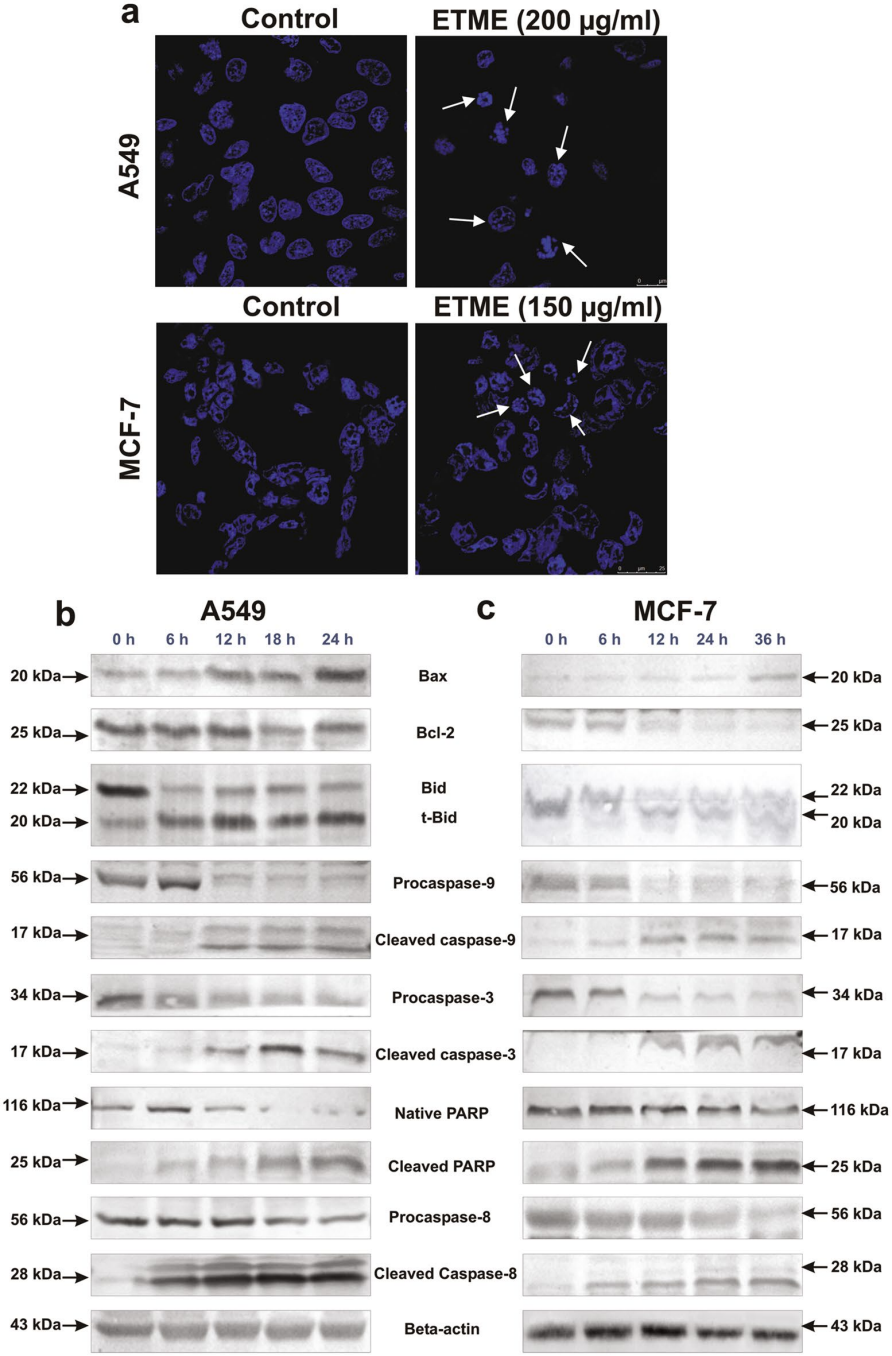
Observation of DNA fragmentation and westerblot analysis of apoptotic proteins in ETME treated *A549* and *MCF-7* cells. **a** The fragmented DNA of nuclei were bind with DAPI and observed under a confocal microscope (630×). The *white arrows* indicate cells with fragmented DNA of nucleus after the treatment of ETME. **b** Effect of ETME on apoptotic proteins in *A549* (200 µg/ml) cells and **c** *MCF-7* (150 µg/ml) cells analysed by immunoblotting after various time intervals. Expression of β-actin was set as a protein loading control

We next investigated whether ETME could induce apoptosis in both A549 and MCF-7 cells. After 48 h of ETME treatment the percentage of apoptotic cells increased, from 0.13 % in the control to 71.36 % (200 μg/ml) in case of A549 (Fig. 2e) and from 0.46 % in the control to 94.65 % (200 μg/ml) in MCF-7 cells (Fig. 2f). Both the untreated and ETME (200 μg/ml for A549 and 150 μg/ml for MCF-7) treated cells were stained using DAPI and pictures were captured by confocal microscopy (Fig. 3a). Untreated cells were found with normal morphology and intact nuclei, while the treated cells exhibited the characteristics of apoptosis, with condensed and fragmented nuclei.

### ETME regulates the Bax/Bcl-2 ratio and activates caspases in both A549 and MCF-7 Cells

To know the underlying mechanism responsible for ETME-induced apoptosis, we next examined numerous proteins that are involved in the regulation of apoptosis in A549 and MCF-7 cells by Western blot analysis. We found that ETME upregulated the expression of Bax and downregulated the expression of Bcl-2 in a time-dependent manner, leading to increase in the Bax/Bcl-2 ratio in both cells. Activation of different caspases like caspase-3, caspase-8, caspase-9, truncation of BID and degradation of PARP after 12 h of ETME treatment (Fig. 3b, c) in both A549 and MCF-7 cells, are hallmarks of apoptosis.

### Effect of ETME on cell migration

**Fig. 4:**
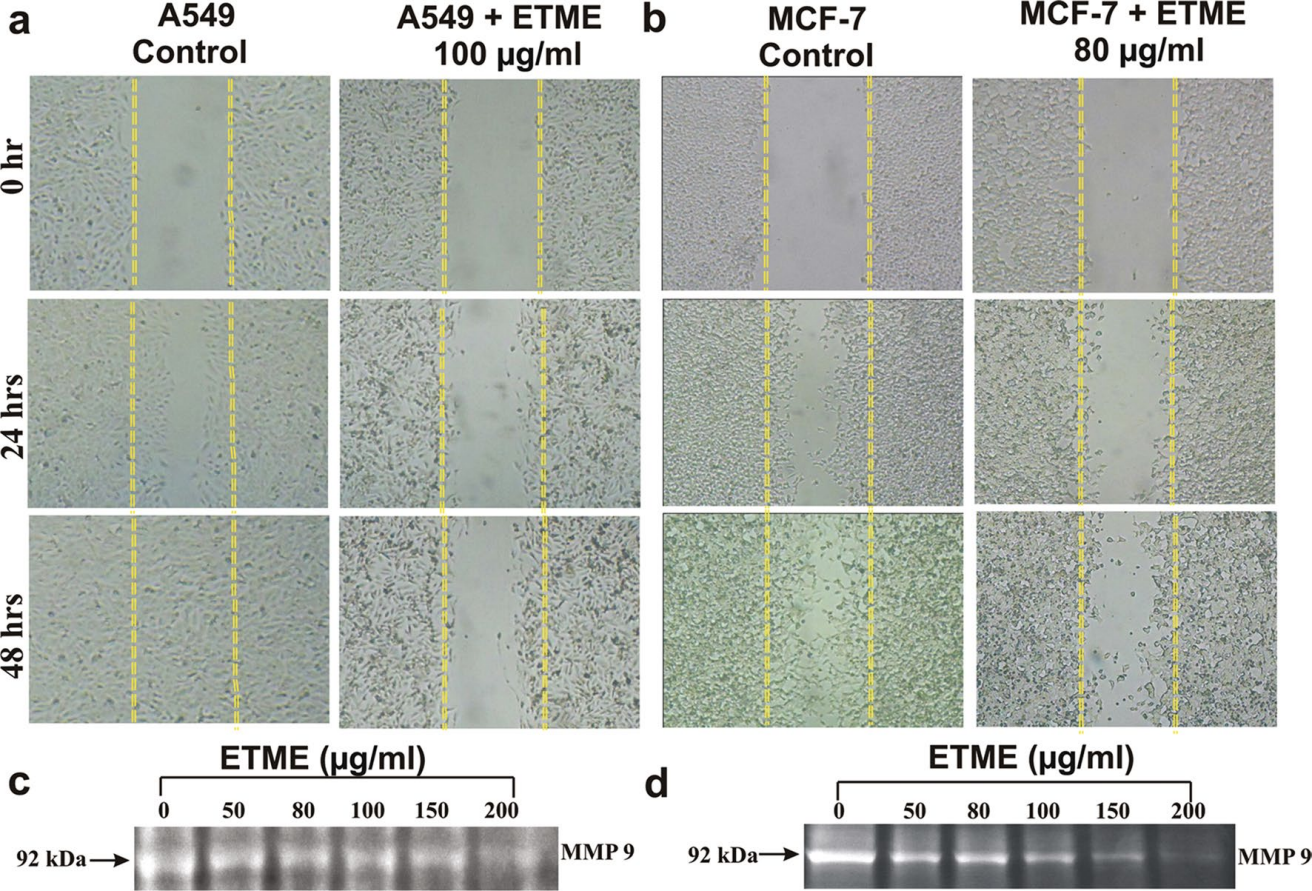
Wound healing activity of untreated and treated *A549* and *MCF-7* cells observed under a light microscope (100×). **a** Representative cell migration images of wounded *A549* cells monolayer treated with ETME; **b** Representative cell migration images of wounded *MCF-7* cells monolayer treated with ETME. **c**, **d** Effect of ETME on the secretions of MMP-9 in *A549* and *MCF-7* cells observed by gelatin zymography

The effect of ETME on A549 and MCF-7 cell migration was determined by scratch motility assay. After 48 h, both the untreated A549 and MCF-7 cells displayed a complete wound closure activity (Fig. 4a, b). In contrary, ETME showed a decreasing migration of A549 and MCF-7 cells in a time-dependent study using the low dose which was confirmed by cell viability assay (100 μg/ml for A549 and 80 μg/ml for MCF-7). Additionally, we investigate the inhibitory effect of ETME on the upregulation of MMP-9 through gelatin zymography. As shown in Fig. 4c, d, MMP-9 expression was inhibited by ETME with increasing dose. We found that the expression of MMP-9 decrease after treatment with 100 μg/ml in A549 and 80 μg/ml in MCF-7 cells.

### ETME increases ROS levels in both cancer cells, not in normal cells

**Fig. 5:**
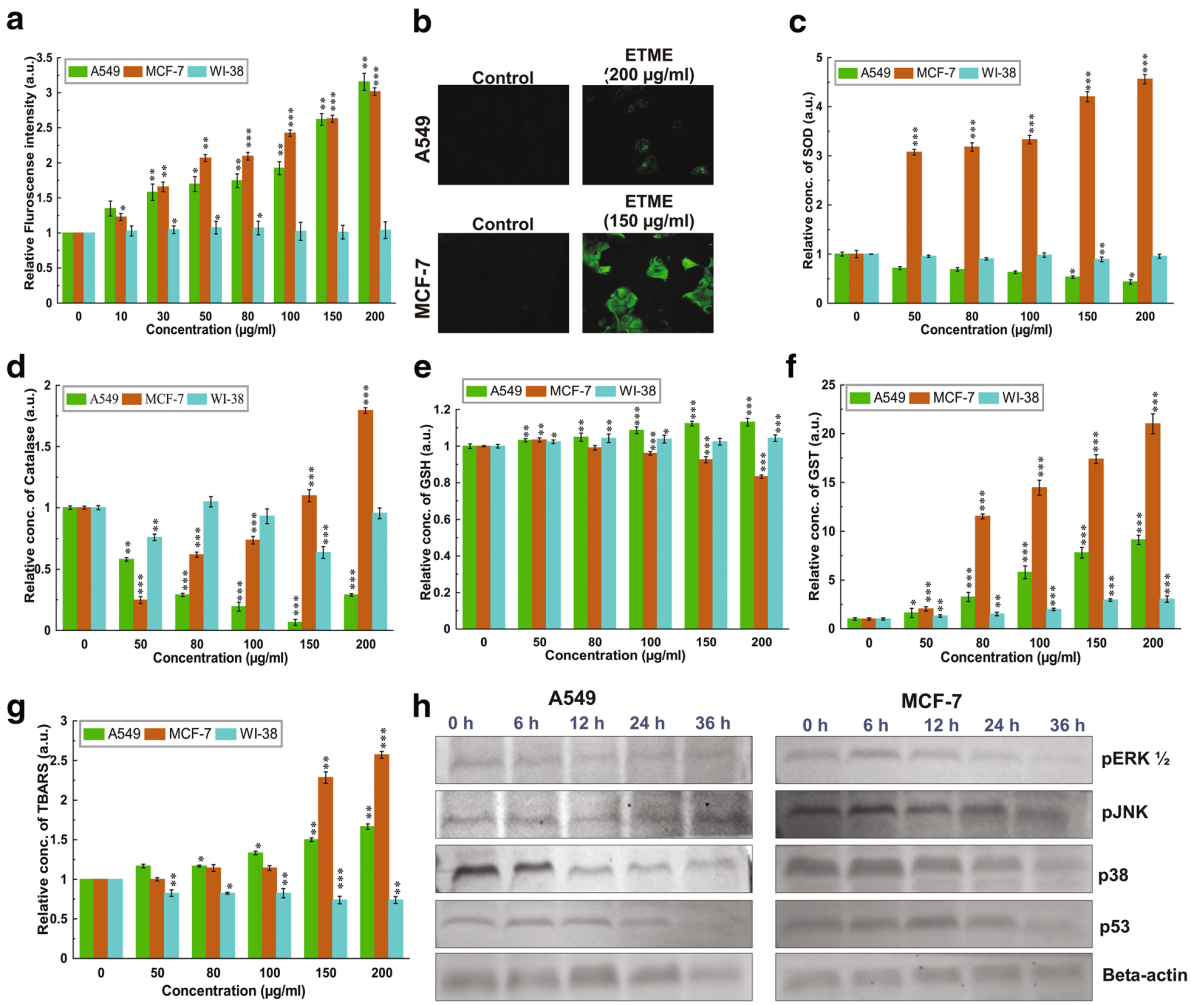
ETME induces ROS by regulating intracellular antioxidants with suppression of MAPK pathways. ETME stimulates ROS generation in A549 and MCF-7 cells, not in normal WI-38 cells. **a** Intracellular ROS levels were examined under FACS using the DCFH-DA staining method and presented graphically; **b** Intracellular ROS was also visualised under the confocal microscope with 400× magnification; ETME regulate levels of major antioxidants such as **c** SOD, **d** catalase, **f** GST, **e** GSH and **g** TBARS in A549 and MCF-7 cells compared to normal WI-38 cells. All data is expressed as mean ± SD (n = 6). *p < 0.05, **p < 0.01 and ***p < 0.001 vs. 0 µg/ml; **h** Western blot analysis of MAPKs and p53 of A549 and MCF-7 cells treated with 200 and 150 µg/ml of ETME respectively. Expression of β-actin was set as a protein loading control

To examine whether ETME has any effect on ROS level in A549, MCF-7 and WI-38 cells, we used fluorescent dye (DCFH-DA) to determine the intracellular ROS level in the presence or absence of ETME. As shown in Fig. 5a, both cells that were treated with 0-200 μg/ml ETME for 24 h had remarkably increasing ROS levels than normal WI-38 cells. ROS levels were showed to be increased by 1.92 (100 μg/ml), 2.62 (150 μg/ml) and 3.15-folds (200 μg/ml) in case of A549 cells and 2.42 (100 μg/ml), 2.63 (150 μg/ml) and 3.01-folds (200 μg/ml) in case of MCF-7 cells by ETME over that in untreated cells. In confocal microscopy study, the significant stimulation of ROS was also observed by ETME in A549 and MCF-7 cells (Fig. 5b), it means that the stimulation of ROS by ETME could be specific to both cancer cells.

### ETME regulates the levels of intracellular antioxidants

To determine whether ETME has any effect on antioxidant enzymes level in A549, MCF-7 and WI-38 cells, we checked the levels of these proteins along with GSH and TBARS. From Fig. 5c–g we found that SOD (4.56 fold), catalase (1.79 fold), GST (21 fold) and TBARS (2.57 fold) level increased in MCF-7 cells and GSH (1.13 fold), GST (9.13 fold), TBARS (1.67 fold) level increased in A549. Remarkably, ETME suppresses the enzyme expression of SOD (0.6 fold), CAT (0.8 fold) in A549 cells and GSH (0.2 fold) level in MCF-7 cells. Beside slight change in GST level, all the other antioxidants remain unchanged in WI-38 cells.

### Role of MAPK in ETME-induced ROS

Numerous anticancer compounds promote ROS production and regulate MAPK signalling and finally cause apoptosis [24]. We next studied the phosphorylation (activation) status of the ERK 1/2, p38 MAPK, JNK and expression level of tumour suppressor p53. Both A549 and MCF-7 cells were exposed to ETME for several time lengths (0–36 h) and the activations of the ERK 1/2, p38 MAPK and JNK pathways were determined by western blot analysis. The levels of pERK 1/2, p-p38 MAPK, pJNK and p53 were gradually decreased in A549 cells (Fig. 5h) and the same are also downregulated in MCF-7 cells except p53 (Fig. 5h). In case of MCF-7 cells, p53 is upregulated up to 12 h followed by downregulation in case of further incubation.

### ETME interacts with DNA and protein

**Fig. 6:**
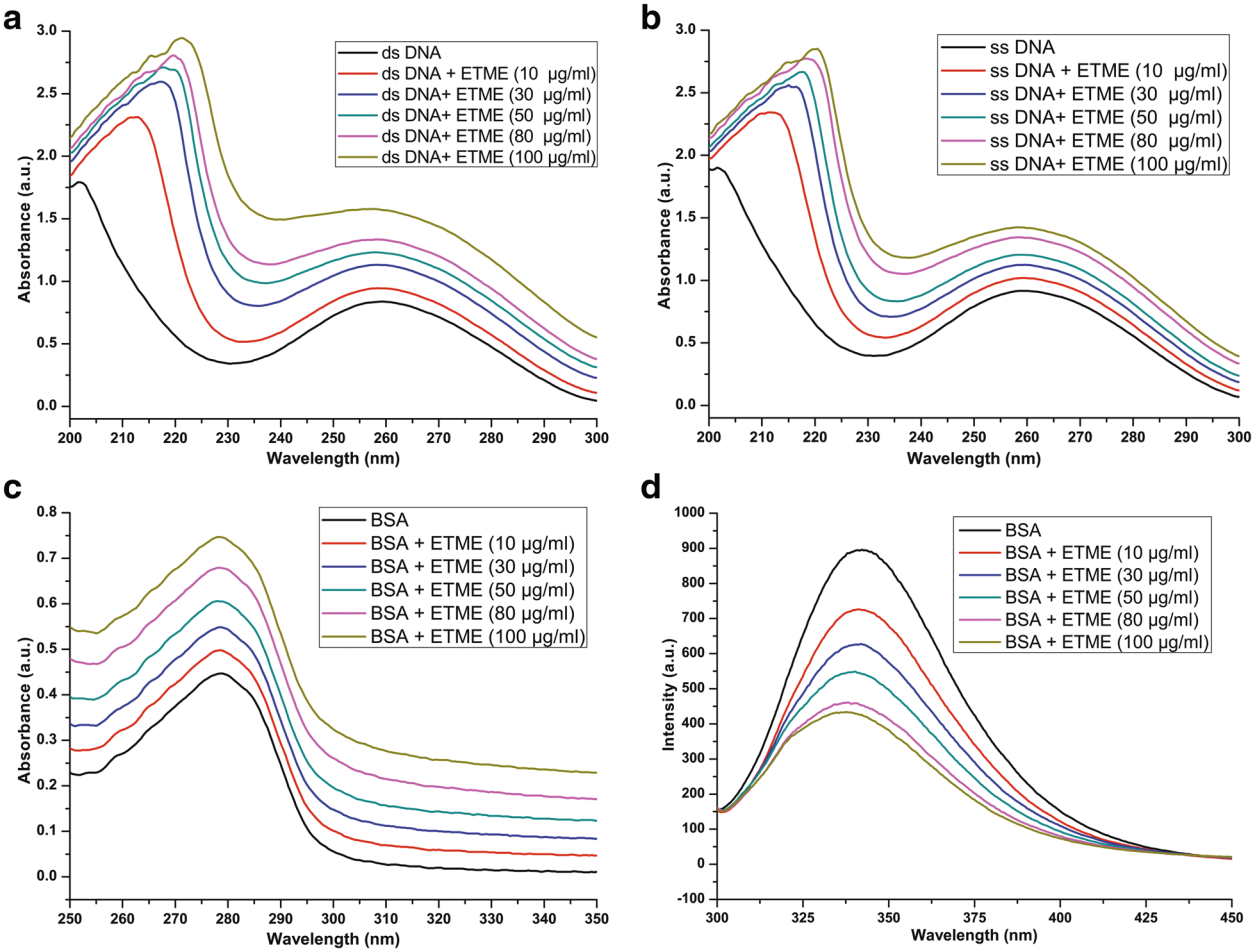
UV visible spectra obtained with increasing concentrations of ETME interaction with Salmon sperm DNA. **a** dsDNA + ETME under native conditions (25 °C) and **b** ssDNA + ETME under denaturing conditions (95 °C). **c** UV-visible spectra obtained with increasing concentrations of ETME interaction with BSA protein at 25 °C. **d** Fluorescence spectra of BSA with various amounts of ETME (pH 7.4, 25 °C). λex = 288 nm, λem = 280–500 nm. Conditions of each spectra are marked with appropriate numbering

The UV–vis absorption spectra of ds DNA and denatured DNA (ss DNA) in the presence and absence of ETME are illustrated in Fig. 6a, b. It was observed that the absorbance (215 and 260 nm) elevated by increase in ETME concentration in both cases of DNA.

In case of UV–vis absorption spectra BSA possessed two absorption peaks at 220 and 280 nm, as shown in Fig. 6c and BSA possessed one soret peak at 340 nm in fluorescence spectra (Fig. 6d). In case of UV–vis absorption spectra, the increasing trend of absorption peaks was observed with increasing concentration of ETME. In addition in fluorescence spectra the decreasing trend also follows.

### Synergistic effect of bioactive compounds present in ETME

**Fig. 7:**
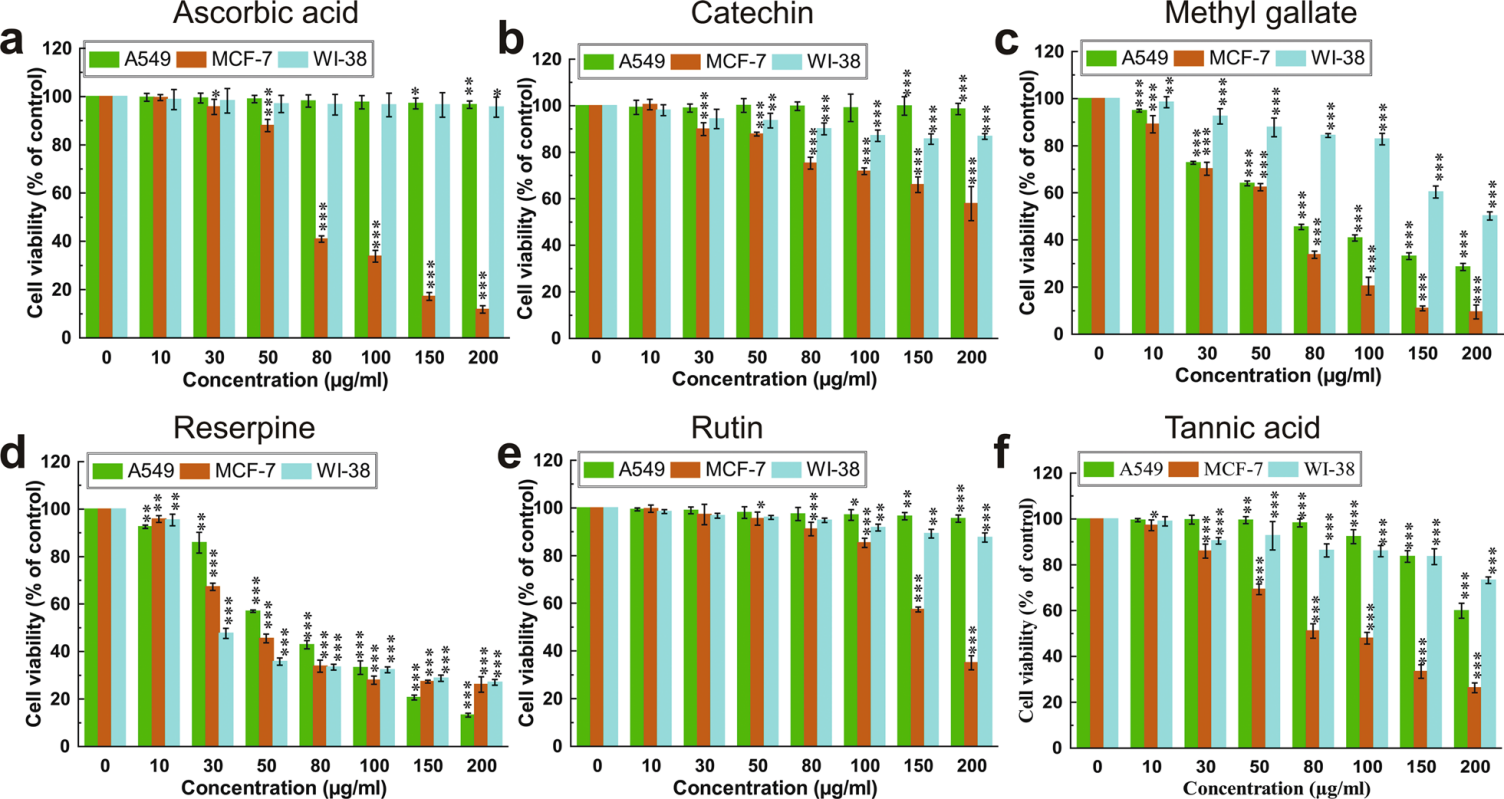
Effect of various bioactive compounds such as **a** ascorbic acid, **b** catechin, **c** methyl gallate, **d** reserpine, **e** rutin and **f** tannic acid present in ETME on cell viability and growth of A549, MCF-7 and WI-38 cells using WST-1 assay. Results were expressed as cell viability (% of control). All data is expressed as mean ± SD (n = 6). *p < 0.05, **p < 0.01 and ***p < 0.001 vs. 0 µg/ml

Previously, we reported that higher quantity of flavonoids, with considerable quantities of alkaloids, phenolics, carbohydrates, tannins and little quantity of ascorbic acid were present in ETME. Moreover, presence of probable active compounds such as ascorbic acid, catechin, methyl gallate, reserpine, rutin and tannic acid were detected through HPLC [22]. The cytotoxicity of these compounds was investigated individually against A549, MCF-7 and WI-38 cells. It was observed that tannic acid, reserpine and methyl gallate showed promissive cytotoxicity against A549, MCF-7 and WI-38 cells. Moreover, ascorbic acid, rutin, catechin showed negligible cytotoxicity towards A549 and WI-38 cells while found toxic against MCF-7 cells (Fig. 7; Table 1). ETME, on the other hand, showed selective cytotoxicity against A549 and MCF-7 cells while being non-toxic to normal cells.

**Table 1:** IC_50_ values of the identified compounds and ETME against A549, MCF-7 and WI-38 cells

Sample	A549	MCF-7	WI-38
Ascorbic acid	6018.19 ± 3435.95	79.14 ± 2.2	6802.54 ± 2248.78
Catechin	4010.65 ± 1849.57	256.52 ± 17.14	753.56 ± 31.99
Methyl gallate	77.25 ± 1.82	53.05 ± 1.91	344.1 ± 31.34
Reserpine	68.46 ± 1.73	57.26 ± 2.29	50.47 ± 1.48
Rutin	4175.01 ± 1608.96	271.3 ± 9.85	1230.51 ± 195.42
Tannic acid	302.25 ± 11.86	97.24 ± 4.89	308.32 ± 8.32
ETME	92.14 ± 6.57	50.27 ± 1.6	911.43 ± 212.2

All the IC_50_values are determined in µg/ml. Data expressed as mean ± SD (n = 6)

## Discussion

Algae are an important source of different bioactive compounds such as antioxidants, antivirals, antimicrobials etc. [25]. Many algae have shown cytotoxic and antitumor activities against different types of cancers [26]. These antitumor activities can play a significant role in development of new pharmaceutical compounds as antitumor drugs [27]. In our study, ETME had been considered for its probable anticancer activity against two kinds of human tumor cell lines: A549, MCF-7. Results from this study demonstrated that ETME inhibited the growth of A549 and MCF-7 cells through the induction of apoptosis, while showed negligible cytotoxicity towards non-malignant cells (WI-38). ETME promoted apoptosis in both type of cells with an effective dose 200 μg/ml for A549 and 150 μg/ml for MCF-7. Further, morphological study from DAPI staining also supported that the above-mentioned doses of ETME are effective in inducing apoptosis in both types of cells.

In the modulation of apoptotic signalling pathways, caspases play important roles in cells and hence their survival and death. Moreover, the fate of cells towards survival or death relies on critical balance between Bcl-2 (antiapoptotic) and Bax (pro-apoptotic) proteins. Time-dependant increase of Bax/Bcl-2 ratio after the treatment of ETME resulted in apoptosis through intrinsic pathway. ETME treatment further led to the downregulation of pro-caspase 8 and upregulation of its active form along with formation of t-Bid on ETME treatment. Caspase 8 has been known to connect intrinsic and extrinsic apoptotic pathways via Bid cleavage to t-Bid, thus suggesting its importance in activation of both the pathways [28]. These results suggest that ETME regulates various proteins involved in intrinsic and extrinsic pathways thereby inducing apoptosis. Ineffectiveness of presently existing treatments is generally due to the invasive and metastatic abilities of malignant cancer cells. Numerous studies have established that suppression of these stages results in the prevention of metastasis and they are the targets of anticancer drug development. We found that ETME-treated cells exhibited downregulated MMP-9 expression in both carcinomas, compared to the control cells. Additionally, a remarkable decrease of cell motility was also seen at a lesser toxic concentration of ETME, signifying its capability to inhibit cell’s motility. This capability of ETME to inhibit cell’s motility can be interconnected with its ability to inhibit the invasiveness of cells since suppression of MMP-9 activities had also been found to be able to reduce migration of cells. Evidences proposed that various natural compounds, for example curcumin and resveratrol, behave either as antioxidant or pro-oxidant depending on the concentration used and the aimed cells [29, 30]. Neferine could trigger mitochondrial-mediated ROS formation in inducing apoptosis in HepG2 cells [31]. Here, we observed that while ETME had an effective cytotoxicity on lung and breast cancer cells, it showed a negligible effect on normal human fibroblast cells. At the same time, our result suggested that ETME significantly increased enormous ROS generation in both cancer cells, but failed to generate in WI-38 cells. A549 cells treated with ETME displayed increase in levels of GST and GSH as well as production of TBARS (cell damaging index) while SOD and CAT activities decreased over 48 h of treatment. In case of MCF-7 cells exposed with ETME, SOD, CAT, GST activity and TBARS production is increased, while GSH level remained unaffected. There is a threshold of ROS above which cells cannot tolerate. A minimum increase of ROS levels in cancer cells can be toxic, making these cells more susceptible to ROS-induced cell death due to the failure of antioxidant defence mechanism [12]. But in case of catalase in A549 cells, probably H_2_O_2_ suppress the transcription factor FoxO1 by PI 3 kinase/Akt-dependent phosphorylation, where PI 3 kinase/Akt signaling is essential for this negative regulation of catalase [32]. We speculated that suppression of SOD gene transcription in A549 cells results possibly in lower activity of SOD leading to increase intracellular ${\text{O}}_{{2^{ - } }}$ levels and furthermore the OH production through Fenton reaction known to initiate lipid peroxidation. Additionally, superoxide ions can prevent GPx activity through oxidative alterations [11]. GSH is an antioxidant and its reduction in intracellular levels in MCF-7 cells are connected with enriched susceptibility to ROS-induced apoptosis [33]. Surprisingly, in normal cells, the balance of antioxidants is maintained.

The MAPK family have important roles in cell survival and death. It is familiar that JNK, p38 and p53 trigger apoptosis while ERK helps cell survival [34]. Previous studies have shown that ERK is specially stimulated by mitogen through the involvement of Ras/Raf/MEK in MAPK pathways, thus leading to cell growth and survival [35]. Furthermore, JNK and p38 MAPK signalling molecules are predominantly activated depending upon the inflammatory cytokines and environmental stress, which ultimately helps in cell differentiation and apoptosis [36]. In this study, we observed a decreased p-ERK1/2, JNK and p38 in response to ETME treatment. In addition, JNK may have a role as an antiapoptotic protein kinase in few tumors. Antisense oligonucleotides, specific to JNK can lead to the reduction of its mRNA level, thereby restricting the growth of A549 cells, probably by promoting apoptosis [37]. Our report showed that phosphorylation of p38 was inhibited dose-dependently by ETME. This discrepancy may be due, at least in part, to the variation of cell type’s. In case of MCF-7 cells p53 was upregulated up to 12 h followed by downregulation after treatment with ETME might be due to the loss of cells indicating that ETME induces apoptosis in MCF-7 cells by activating p53. However, in case of A549 cells time dependent downregulation of p53 expression was observed suggesting ETME induce p53 independent apoptosis in this type of cells. This regulation in MAPK pathways could be responsible for the activation of apoptotic pathways and inhibition of MMPs expression since inhibition of Ras in vitro has been shown to stop MMPs formation [38].

UV spectroscopy is widely adapted as a useful technique for studying drug–DNA and drug–protein interactions. The UV–visible spectra of ds and ss DNA show a prominent absorption peak at 205 nm and slight red shift also observed after addition of increasing concentrations of ETME. Moreover, another peak i.e. 260 nm also increase in its intensity after ETME addition suggesting hyperchromicity [39]. A continuous hyperchromicity of the soret band upon addition of ETME to ds DNA solution may be an indication of electrostatic binding or partial unwinding or intercalative mode of binding involving a strong stacking interaction between an aromatic chromophore and the base pairs of DNA [40–42]. In order to understand the interaction of the ETME with denatured DNA, the DNA-compound mixture was heated at 95 °C for 15 min. The resulting spectra (Fig. 6b) revealed a moderately lower hyperchromic shift than the native conditions. Under denatured conditions the DNA double helix uncoils, exposing more number of nitrogen bases to the medium which loss interface electrostatic interactions with the complexes present in ETME, indicating the presence of DNA intercalating agents in ETME.

In case of protein, UV–vis absorption measurement is a simple and relevant method that is used to investigate structural changes and to discover complex formation [43, 44]. The elevated levels of absorbance after the addition of ETME indicate the formation of a ground state complex. As dynamic quenching does not affect the absorption spectrum of a molecule and it only affects the excited states of a molecule, the observed changes in BSA absorbance in the presence of different concentrations of ETME could be an indicative of occurrence of static quenching interaction between the compounds present in ETME and BSA [45]. Also, fluorescence spectroscopy has been regarded as the most comprehensive method for studying protein–ligand interactions especially in dynamic states [46]. Generally, BSA fluorescence absorption originates from Trp, Tyr and Phe residues, whereas its intrinsic fluorescence can be mainly attributed to the Trp residue alone [47]. The fluorescence intensity of BSA was decreased dose-dependently with increase in ETME concentrations (Fig. 6d) and no emission spectral shifting was observed, indicating that ETME could interact with BSA, and that the fluorescence chromophores of BSA were not exposed to an obvious polarity change with ETME titration.

It was also described that no single class of compounds in an extract could be completely held accountable for the activity produced by the total extract itself [48]. Our result suggested that probable bioactive compounds present in ETME, individually are not specific to kill cancer cells, they also affected the growth of normal cells. Therefore, it is more significant as well as prudent to assess the activity of ETME as a complete mixture of interacting bioactive compounds rather than evaluating them as a breakup of their individual components.

## Conclusions

Our findings indicated that mixture of bioactive compounds present in ETME effectively inhibits the growth of A549 and MCF-7 cells without hampering the normal WI-38 cells. ETME induces the apoptosis and suppresses metastasis including cell migration and cell invasion through the elevation of intracellular ROS levels following suppression of MAPK pathway in both the cells.

## Methods

### Collection, extract and sample preparation of microalga

Microalgal sample were gather from various ponds in the Bankura district in the state of West Bengal, India in April 2014. This algal sample was authenticated by Dr. R. K. Gupta, Botanical Survey of India, Kolkata, India and preserved in formalin (4 %) for identification and noticed under the light microscope using standard methods [49]. The procedure of extract preparation of *E. tuba* followed exactly is same like previous [23]. The microalgal extract was taken and dissolved in sterile distilled water followed by filter sterilization using 0.22 μm syringe filter and a stock of 2 mg/ml was prepared for conducting all experiments.

### Cell lines and culture

Human lung adenocarcinoma (A549), human breast adenocarcinoma (MCF-7) and human lung fibroblast (WI-38) cell line were obtained from the National Centre for Cell Science (NCCS), India and kept in the ideal laboratory condition. A549 cells were cultured as monolayers in Ham’s F-12 medium whereas MCF-7 and WI-38 cells were cultured as monolayers in DMEM. Both the media were supplemented with 10 % (v/v) fetal bovine serum (FBS), 100 U/ml Penicillin G, 100 µg/ml Streptomycin, 50 µg/ml Gentamycin sulphate and 2.5 µg/ml Amphotericin B. All the cell lines were kept in a humidified atmosphere containing 5 % CO_2_ in incubator at 37 °C and passaged tri-weekly.

### Determination of cytotoxicity using WST-1 Assay

Cell viability were analysed using the WST-1 cell proliferation reagent according to the earlier described technique [50]. All cells (1 × 10^4^ cells/well) were treated for 48 h with concentrations ranging from 0–200 µg/ml of ETME, ascorbic acid, catechin, tannic acid, reserpine, methyl gallate and rutin. Cell proliferation and viability were measured by measuring absorbance of the coloured product spectrophotometrically at 460 nm using a microplate ELISA reader MULTISKAN EX (Thermo Electron Corporation, USA).

### Cell cycle analysis

According to a previously described method, cell cycle analysis was studied by flow cytometry [50]. Cell cycle phase distribution of treated and untreated cells was examined by flow cytometry [FACS Verse (Becton–Dickinson) equipped with 405 nm (Violet), 488 nm (Blue) and 640 nm (Red) solid state laser light using FACSuite software Version 1.0.3.2942] by acquiring at least 10,000 cells per sample. The percentage of populations in the G0/G1, S and G2/M phases were determined using similar software.

### Annexin V/propidium iodide staining

Apoptotic cells were quantified by Propidium iodide (PI) and Annexin V-FITC double staining, using an Annexin-V-FLUOS Staining Kit, Roche Diagnostics according to manufacturer’s protocols. Treated and untreated cells were stained with PI and FITC according to the manufacturer protocol. The distribution of apoptotic cells was quantified by flow cytometer. A total 10,000 events were acquired.

### DAPI (4′,6′-diamidino-2 phenylindole) staining

According to an earlier described method, cell morphology was observed by DAPI staining [51]. The undergoing apoptosis cells, represented by the morphological alteration of apoptotic nuclei, were observed and taken image from ten eye views at 630× magnifications under a laser scanning confocal microscope Leica TCS SP8 (Leica, Illinois, United States) and the data was analyzed using Leica Application Suite X software.

### Immunoblot analysis

Both A549 and MCF-7 cells were treated with 200 and 150 µg/ml ETME respectively for different time intervals (6–36 h) for testing apoptotic and MAPK related protein study. Protein was quantified by the Folin-Lowry method. Equal quantities of proteins (40 mg for apoptotic related and 30 mg for MAPK) were taken for gel electrophoresis. The blot was developed using the previous protocol [51]. The photographs were taken using imaging system EC3 Chemi HR (UVP, USA). The developed blots were then analysed for densities of bands by ImageJ 1.45 s software.

### Scratch motility assay

Both malignant cells were seeded and grown for overnight to make confluent. The monolayer was then scratched vertically using pipette tip followed by washing with PBS two times to eliminate detached cells and incubated with media containing extracts at their respective less-toxic dose for 48 h. After incubation, cells migration on these scratched zones was pictured at five randomly selected fields.

### Gelatin zymography assay

MMP-9 activity was checked by gelatin zymography as described previously [52]. After 48 h treatment with several concentrations of ETME (0–200 µg/ml) both A549 and MCF-7 cells were harvested, total protein was isolated and gelatin gymography was performed. Appearance of unstained bands on a blue background, indicates gelatinolytic activity.

### Measurement of intracellular ROS using DCFH-DA staining

For DCFH-DA staining treated and untreated cells were washed with PBS and loaded with 20 µM DCFH-DA diluted in clear serum free media for 30 min at 37 °C. After that cells were washed twice with PBS and intensity of the intracellular ROS was measured by FACS in all three cell lines and also morphology of cells (A549 and MCF-7 cells) was photographed (400× magnifications) using a previously mentioned laser scanning confocal microscope.

### Measurement of antioxidants by different biochemical assays

After ETME treatment, all the cells were harvested and washed with PBS. The cell pellet was suspended in cold PBS and were lysed on ice using a sonicator and centrifuged at 13800*g* for 20 min at 4 °C. The supernatant was taken for testing antioxidant enzyme assays, such as SOD, CAT, GST and also measuring the production of GSH and TBARS. SOD as checked by measuring the inhibition of the formation of blue colour formazan at 560 nm with slight modification from a previous study [53]. CAT activity was measured with modified protocol continuous time course decaying of H_2_O_2_ at 240 nm [54]. Earlier described method was followed to examine glutathione-*S*-transferase GST based on the formation of GSH-CDNB conjugate [55]. The reduced glutathione (GSH) level was determined spectrophotometrically at 412 nm according to the standard protocol [56]. The level of lipid peroxide in cell homogenates were determined in conditions of thiobarbituric acid reactive substances (TBARS) as an index of accumulation of malondialdehyde [57]. All biochemical tests were assayed in hexaplate.

### DNA and protein binding studies

DNA and protein binding characteristics of the compounds present in ETME were investigated by UV–Visible spectroscopy using a Shimadzu UV-2401 PC UV–VIS recording spectrometer (Shimadzu Corporation, Kyoto, Japan) according to previously described method [58]. In addition, for protein binding study the fluorescence emission spectra was also measured at 25 °C. Spectra were recorded in the wavelength range of 200–500 nm setting the excitation at 288 nm, and emission at 340 nm according to the previously described method [59].

### Statistical analysis

All spectrophotometric data were presented as the mean six times ±SD and cell cycle and ROS analysis data by FACS was introduced as the mean three times ±SD. The statistical analysis was determined by KyPlot version 2.0 beta 15 (32 bit). When A1 = IC_50_, Y = response (Y = 100 % when X = 0), X = inhibitory concentration, then IC_50_ values were calculated by the following formula, ${\text{Y}} = {{100 * {\text{A}}1} \mathord{\left/ {\vphantom {{100 * {\text{A}}1} {\left( {{\text{X}}\text{ + }{\text{A1}}} \right)}}} \right. \kern-0pt} {\left( {{\text{X}}\text{ + }{\text{A1}}} \right)}}$. The IC_50_ values were compared by paired *t* test. P < 0.05 was considered significant.
